# Making “cold” tumors “hot”- radiotherapy remodels the tumor immune microenvironment of pancreatic cancer to benefit from immunotherapy: a case report

**DOI:** 10.3389/fimmu.2023.1277810

**Published:** 2023-12-20

**Authors:** Fan Tong, Yi Sun, Yahui Zhu, Huizi Sha, Jiayao Ni, Liang Qi, Qing Gu, Chan Zhu, Wenjing Xi, Baorui Liu, Weiwei Kong, Juan Du

**Affiliations:** ^1^ Department of oncology, Nanjing Drum Tower Hospital, Affiliated Hospital of Medical School, Nanjing University, Nanjing, China; ^2^ The Comprehensive Cancer Center of Drum Tower Hospital, Clinical College of Traditional Chinese and Western Medicine, Nanjing University of Chinese Medicine, Nanjing, China; ^3^ National Institute of Healthcare Data Science, Nanjing University, Nanjing, China; ^4^ State Key Laboratory of Neurology and Oncology Drug Development Jiangsu Simcere Diagnostics Co, Ltd, Nanjing, China

**Keywords:** immune checkpoint inhibitors, tumor immune microenvironment, radiotherapy, metastatic pancreatic cancer, second-line treatment

## Abstract

Immune checkpoint inhibitors have limited efficacy in metastatic pancreatic cancer due to the complex tumor immune microenvironment (TIME). Studies have shown that radiotherapy can cause cell lesions to release tumor antigens and then take part in the remodeling of the tumor environment and the induction of ectopic effects *via* regional and systemic immunoregulation. Here, we reported a case of advanced metastatic pancreatic cancer treated with immunotherapy combined with chemotherapy and radiotherapy and a sharp shift of the TIME from T3 to T2 was also observed. One hepatic metastasis within the planning target volume (PTV) was evaluated complete response (CR), the other one was evaluated partial response (PR) and 2 hepatic metastases outside the PTV were surprisingly considered PR. In the study, we found that immunotherapy combined with chemotherapy and radiotherapy achieved significant therapeutic benefits, which may provide a new strategy for the treatment of advanced pancreatic cancer.

## 1 Introduction

Pancreatic cancer has very poor prognosis with a 5-year survival rate of only 8% (1). About 50% of patients with pancreatic cancer are diagnosed at an advanced stage (2) and there is no clear consensus on the second-line treatment when first-line treatment based on gemcitabine fails.

In recent years, immune checkpoint inhibitors (ICIs) have achieved decisive breakthroughs in many solid tumors (3–5), but the efficacy of ICIs in pancreatic cancer is still confronted with challenges. The complex TIME of pancreatic cancer limits the effectiveness of ICIs (6), but more and more clinical studies and experiments have proved that radiotherapy combined with immunotherapy can regulate the TIME, so as to strengthen the control of tumor (7, 8).

Here, we presented an advanced pancreatic cancer case with robust survival benefit from immunotherapy combined with chemotherapy and radiotherapy, while obvious TIME remodeling and an ectopic effect were also observed. Briefly, this comprehensive treatment mode remodulated pancreatic cancer from “cold” tumors to “hot” tumors in our case.

## 2 Case presentation

**Figure 1:**
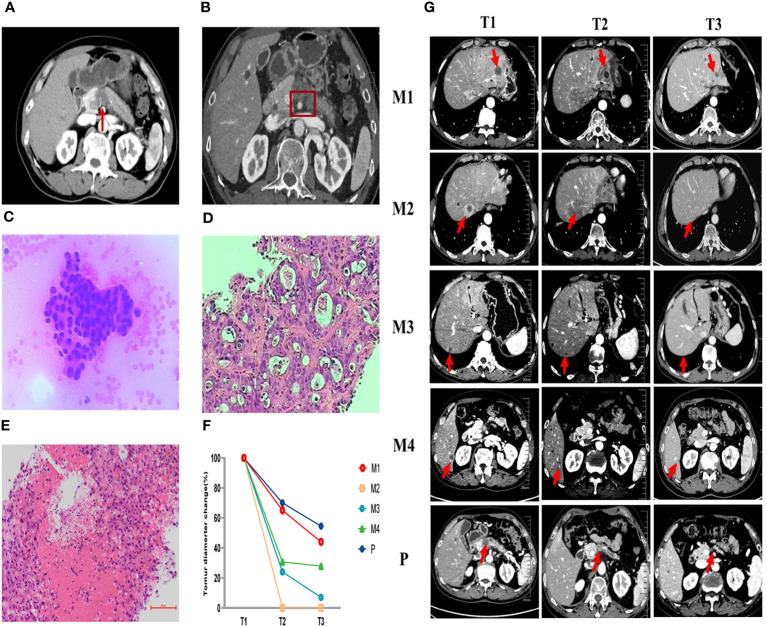
Pathological and imaging evaluations during the first-line (AG) and second-line (SOXPR) treatment. **(A, B)** A 2.2cm × 2cm mass at the neck of the pancreas was detected with superior mesenteric artery (SMA) invasion less than 180° on abdominal CT at baseline. **(C)** Cancer cells were observed in pancreas biopsy (×200) at T0. **(D)** Ultrasound guided biopsy of the hepatic mass (M2) was concordant with liver metastasis of pancreatic ductal adenocarcinoma (×200) at T1. **(E)** Pathology of M2 showed inflammatory cells and no residual tumor cells (×200) at T2. **(F, G)** Abdominal CT during second-line (SOXPR) treatment and the tumor diameter variation. T0, The baseline prior to first-line therapy; T1, The baseline prior to second-line therapy; T2, Obvious relief of TEN symptoms; T3,: Three additional cycles of SOX to end; M1-M4, 4 hepatic metastases; P, primary pancreatic cancer locus.

We presented a case of a 59-year-old male who was hospitalized with intermittent upper abdominal pain in October 2021. Contrast-enhanced computed tomography (CT) scan showed a 2.2cm x 2cm mass at the neck of the pancreas with distal pancreatic duct dilatation (**Figure 1A**). The mass was closely related to the splenic vein. But after discussion, the Multiple Disciplinary Team (MDT) believed that the patient was also accompanied by superior mesenteric artery (SMA) invasion less than 180° (**Figure 1B**). But no distant metastasis was detected at that time. In addition, the baseline value of carbohydrate antigen 19-9 (CA19-9) was 12.99 U/ml. Endoscopic ultrasound-guided fine-needle aspiration (EUS-FNA) was performed and subsequently cancer cells were verified pathologically (**Figure 1C**). The patient was definitely diagnosed with borderline resectable pancreatic cancer based on pathology and imaging. But the patient refused to consider the possibility of follow-up operation firmly at the very start.

From November 2021 to March 2022, the patient received 5 cycles (21 days for one complete cycle) of gemcitabine 1000 mg/m2 and nab-paclitaxel 125 mg/m2 on day 1 and day 8 and the patient stayed a stable disease. After 5 cycles of the treatment, CA19-9 increased to 76.7U/ml. In addition, CT scan revealed that the size of pancreatic primary tumor had increased remarkably and four new hepatic masses appeared (**Figure 1G-T1**). Pathology for Ultrasound guided biopsy of the hepatic mass was concordant with liver metastasis of pancreatic ductal adenocarcinoma (**Figure 1D**). The patient was assessed progressive disease (PD).

**Figure 2:**
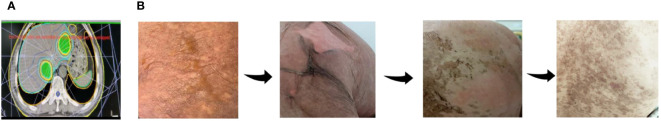
Occurrence of TEN after SOXPR. **(A)** Planning target volume of hepatic metastases radiotherapy. **(B)** Skin changes during treatment of TEN.

Subsequently, S-1 plus oxaliplatin combined with immunotherapy and radiotherapy were used in second-line treatment. In detail, the patient received 3 cycles of S-1 80mg/day on day1-14 plus oxaliplatin 130mg/m2 on day 1 and Sintilimab 200mg on day 1 (21 days for a complete cycle) while 8Gy*3 fractions radiotherapy of liver metastases within PTV was conducted before Cycle2 started (**Figure 2A**).

After finishing 3 cycles of this treatment, the patient developed toxic epidermal necrolysis (TEN) and after the Multiple Disciplinary Team (MDT) discussion, the experts unanimously assessed TEN as immune-related adverse event (irAE). After methylprednisolone, anti-infection, fluid infusion treatment, his symptoms quickly relieved (**Figure 2B**) and tumor marker CA19-9 decreased to 19.2 U/ml by the TIME. CT scan revealed that the primary pancreatic tumor and hepatic metastases had both shrunk remarkably (**Figure 1G-T2**). Surprisingly, a hepatic metastasis within the scope of radiotherapy had disappeared in CT scan. Obvious inflammatory cell infiltration was confirmed by pathology and no cancer cells was found in the biopsy tissues (**Figure 1E**). One hepatic metastasis within the scope of radiotherapy was assessed CR, the other one was evaluated PR, and other two hepatic metastases outside the scope of radiotherapy were also considered PR according to the Response Evaluation Criteria in Solid Tumors (RECIST1.1) criteria.

**Figure 3:**
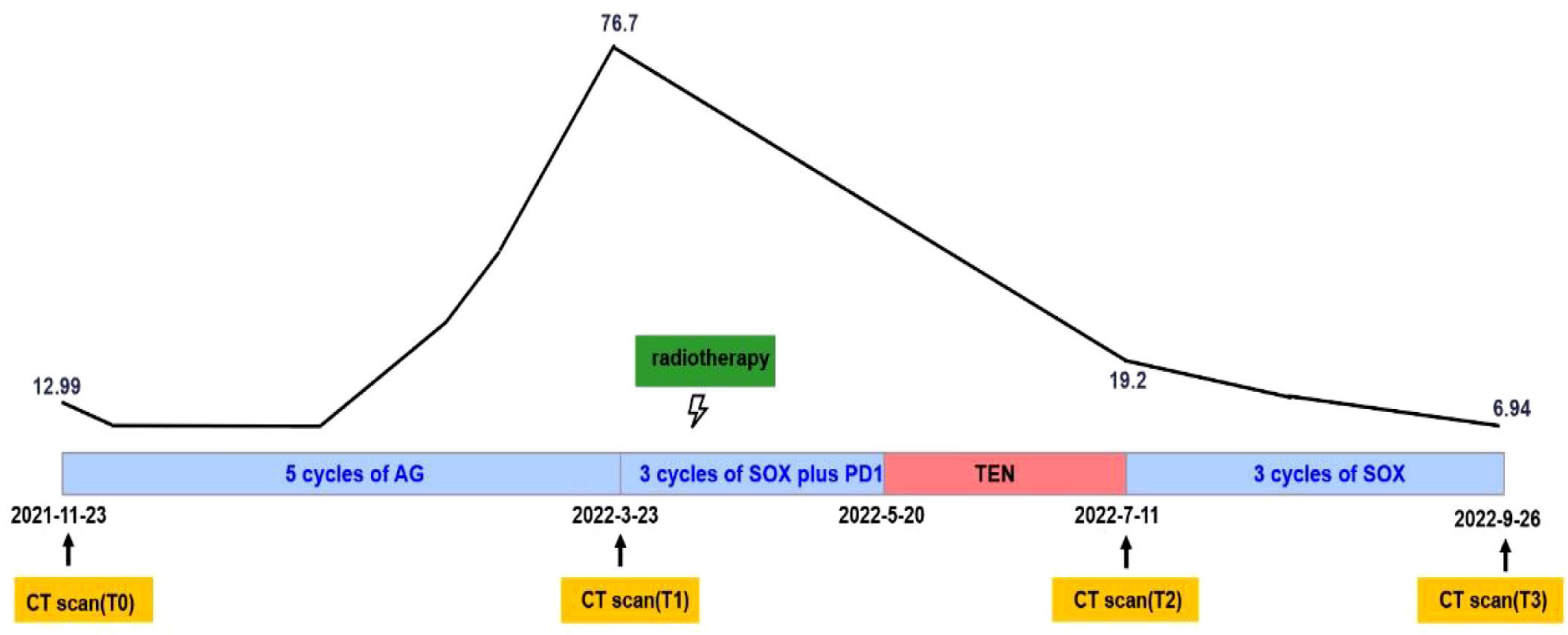
Systemic illustrations of clinical therapy flow chart. Broken Line indicates CA19-9 levels of this patient during the treatment.

We conducted the SOX regimen for another 3 cycles when symptoms related to TEN were greatly relieved. At the time, CA19-9 decreased to 6.94U/ml and all tumors continued to shrink as CT indicated (**Figure 1G-T3**). Tumor diameter changes were demonstrated in **Figure 1F**. The concomitant changes of CA199 and timeline of events were demonstrated in **Figure 3** in details.

**Figure 4:**
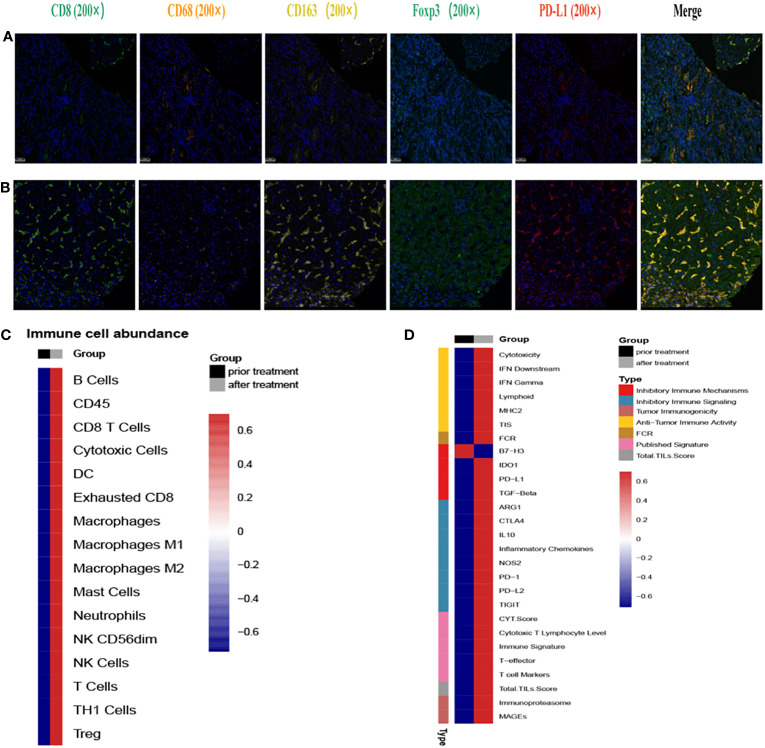
Immunofluorescence and RNA immune-related panel sequencing prior and post second-line treatment. **(A)** The representative immunofluorescent images of CD8, CD68, CD163, FoxP3 and PD-L1 of tumor tissues prior second-line treatment. **(B)** The representative immunofluorescent images of CD8, CD68, CD163, FoxP3 and PD-L1 of tumor tissues post second-line treatment. **(C)** Immune cell-related gene expression prior and after second-line treatment. Black indicates pre-treatment, gray indicates post-treatment; blue indicates decreased expression, red indicates increased expression. **(D)** Immune signature-related gene expression prior and after second-line treatment. Black indicates pre-treatment, gray indicates post-treatment; blue indicates decreased expression, red indicates increased expression.

To comprehensively assess the alteration of TIME before and after treatment, we performed multiplexed immunofluorescence histochemical (mIHC) analysis at the protein level and gene expression analysis at the RNA level on M2 hepatic metastasis prior and post treatment, respectively. The spatial immune microenvironments of tumor tissues prior (**Figure 4A**) and post treatment (**Figure 4B**) were shown by mIHC assay. The relative values of CD8+, CD68+, CD163+, Foxp3+, and PD-L1+ were 7.74, 3.9, 1.91, 2.84, and 0.69 respectively, showing a high infiltration of immune cells and low expression of PD-L1 (subtype TIME-3, immune escape type). After 3 cycles of immunotherapy combined chemoradiotherapy, the relative values of CD8+, CD68+, CD163+, Foxp3+, and PD-L1+ were 19.02, 6.06, 30.44, 8.11, and 21.76 respectively, showing a high infiltration of immune cells and high expression of PD-L1 (subtype TIME-2, immune response type). The RNA-level expression assay of TIME was detected by 289 immune-related genes (NanoString Technologies, Seattle, USA) at Jiangsu Simcere Diagnostics Co., Ltd, and the selection of immune-related genes is shown in **Supplementary Materials**. The abundance of immune cells related with the tumor microenvironment was shown in **Figure 4C**, and the abundances of all immune cells were elevated to different degrees, and other immune signatures in **Figure 4D**. For example, the CD8+ T cell score increased from 4.13 to 7.48, and the Macrophage gene score improved from 6.05 to 8.07, and the Treg gene score improved from 3.32 to 5.36. In addition, the mIHC results also revealed an increase in Treg cells and M2 macrophages, consistent with previous study that the effect of radiotherapy on the tumor microenvironment may be dual, inducing both an immunostimulatory effect (recruitment of T cells) and an immunosuppressive effect (expansion of Treg cells) (9). Therefore, we hypothesized that this coexistence of immunostimulatory and immunosuppressive effect in radiotherapy leads to stabilization of the patient’s disease and may provide opportunities for immunomodulation (10). Scores of other signatures or markers also increased, such as the scores of IFNγ from 5.29 to 8.51, cytotoxic T lymphocyte from 4.43 to 7.64. Interestingly, the change in the scores for B7-H3 showed a decreasing trend in contrast to the other scores, and previous studies have also shown a negative correlation between its high expression and treatment response. Additional immune scores were shown in **Supplementary Table 1**. Both mIHC, as well as TIME assays at the RNA level, reveal that immunotherapy combined with chemoradiotherapy enhances immune cell infiltration, which may be responsible for promoting the immune response and benefiting patient’s clinical response.

## 3 Discussion

At present, chemotherapy is still the main treatment for advanced pancreatic cancer. With the deepening understanding of the pathogenesis of pancreatic cancer, immunotherapy based on remodeling TIME has become a hot topic of pancreatic cancer treatment (11). However, the specific and complex TIME of pancreatic cancer limits the effectiveness of immune checkpoint inhibitors therapy (12–15). Studies have shown that nearly 50% of the stroma cellular component of pancreatic cancer tissue is immune-related cells, but only a few are anti-tumor-related effector cells (16). Single-agent immunotherapy rarely works in second-line therapy in advanced pancreatic cancer, but we made breakthroughs and achieved unexpected clinical efficacy by a cocktail therapy consisted of immunotherapy combined with chemotherapy and radiotherapy in our case.

To better predict the response of immunotherapy in solid tumors, researchers divided the TIME into 4 subtypes based on PDL1 expression and the presence of tumor-infiltrating lymphocytes (TILs): T1 (PDL1−, TIL−), T2 (PDL1+, TIL+), T3 (PDL1−, TIL+), and T4 (PDL1+, TIL−) (17). T2 are considered to be the type to better predict the immune response. Radiation upregulated the expression of PD-L1 (8, 18, 19)and increased the infiltration of CD8+ T cells (20, 21), which changed the TIME from type 3 to type 2 in our case. Hot tumors were remodeled in this way to achieve enhanced clinical efficacy.

On one hand, radiation accelerates tumor cell lesions and death to promote the exposure and presentation of tumor associated antigens. On the other hand, high infiltration of CD8+ T cell is influenced by chemokines such as CXCL9 and CXCL10 (22, 23). Radiotherapy induces the production of these chemokines, and promote the recruitment of T cells to tumor tissues (24–26).T cells infiltration and antigens exposure activate T cell response to release IFN- γ and IFN- γ stimulates the upregulation of PD-L1 (27, 28). Besides, radiotherapy can also up-regulate PDL1 by activating cGAS-STING (cyclic guanosine monophosphate-adenosine monophosphate synthase-stimulator of interferon gene) pathway to lay basis for use of ICIs (29, 30). In addition, radiotherapy has been reported to induce normalization of blood vessels to achieve T cell infiltration, but the exact mechanism has not been fully elucidated (31).

In addition, the mIHC results showed an increase in Foxp3+ regulatory T cells (Foxp3+ Treg), which was consistent with previous studies. In bladder and liver cancer, increased accumulation of Treg cells was observed in tumor tissues after radiotherapy, which was shown to be related to radiation-induced Akt pathway activation (32, 33); In prostate cancer, radiotherapy provides a growth and survival advantage for Tregs by inducing TGF-β (34). However, our study hasn’t explored the mechanism by which radiation therapy increases Foxp3+ Treg cells yet, which need to be explained deeply.

In previous studies, some researchers have paid attention to the ectopic effect of radiotherapy, and the specific mechanism of ectopic effect of radiotherapy is attributed to immune effect (35–37). Radiotherapy can induce immune cells to infiltrate into tumor tissue, produce a large number of reactive oxygen species, activate cytotoxic T lymphocytes (CTLs), and lead to apoptosis of tumor cells (38), this was also confirmed by our results of multiple immunofluorescence histochemistry and tumor microenvironment detection. Therefore, we conclude that the synergistic effect of ectopic radiotherapy and immunotherapy enhances the immune response and provides a new therapeutic strategy for advanced pancreatic cancer.

However, not all patients can benefit from radiotherapy combined with immunotherapy. It is well-known that the timing of radiotherapy and the dose of radiotherapy will affect the effect of immunotherapy. There is no consensus of the best time for radiotherapy, but existing studies have found that simultaneous administration of radiotherapy and immunotherapy or timely immunotherapy after radiotherapy is beneficial to the clinical outcome (39, 40). Taking two factors into consideration, we chose to introduce radiotherapy in the middle course of ICIs usage. One point, immunotherapy enhances the tumor’s sensitivity to radiotherapy by cellular pathways. The other point, radiotherapy upregulated PD-L1 to better response to subsequent ICIs. Up to the optimal dose for radiotherapy, studies have shown that both low-dose and high-dose radiotherapy can affect the efficacy of immunotherapy by inflaming tumors, but the reason is not clear (39, 41). Whether it is related to the type of cancer needs to be further explored. Therefore, our patient benefited from synchronous radiotherapy and chemotherapy combined with immunotherapy, and benefited from high-dose radiotherapy((8Gy*3f). Our patient also benefited from the sensitizing effect of radiotherapy on chemotherapeutic drugs. Clinical studies have shown that S-1 and oxaliplatin can be used as radiosensitizers in the treatment of solid tumors (42–44). S-1 can inhibit the repair of radiation-induced DNA damage and oxaliplatin can inhibit DNA replication and transcription.

More and more studies have shown that immune-related adverse events(irAEs) are related to better therapeutic effects (45, 46). Researchers believe that severity of irAEs are bystander effect from activated T cells (47). Thus, patients who experience more severe irAEs may acquire better clinical outcomes, but this conclusion needs to be supported by more clinical data. Our patient developed TEN after treatment with PD1, and CT scan showed a good tumor regression after remission of symptoms.

In conclusion, we provide a potential treatment strategy for the use of immunotherapy combined with chemotherapy and radiotherapy in patients with advanced pancreatic cancer. We consider that this is a typical case that comprehensive treatment mode can convert pancreatic cancer from “cold” tumors to “hot” tumors. More randomized clinical trials are needed to verify the safety and efficacy.

## Data availability statement

The original contributions presented in the study are included in the article/**Supplementary Material**. Further inquiries can be directed to the corresponding authors.

## Ethics statement

The studies involving humans were approved by the Medical Ethics Committee of Drum Tower Hospital Affiliated to Nanjing University Medical School. The studies were conducted in accordance with the local legislation and institutional requirements. The participants provided their written informed consent to participate in this study. Written informed consent was obtained from the individual(s) for the publication of any potentially identifiable images or data included in this article.

## Author contributions

FT: Writing – original draft. YS: Writing – original draft. YZ: Formal analysis, Writing – review & editing. HS: Formal analysis, Writing – review & editing. JN: Writing – review & editing, Data curation. LQ: Data curation, Writing – review & editing. QG: Writing – review & editing, Methodology. CZ: Writing – review & editing, Methodology. WX: Writing – review & editing, Formal analysis. BL: Writing – review & editing, Conceptualization, Methodology. WK: Conceptualization, Writing – review & editing. JD: Conceptualization, Writing – review & editing, Methodology.
