# Supplementary material for: Making “cold” tumors “hot”- radiotherapy remodels the tumor immune microenvironment of pancreatic cancer to benefit from immunotherapy: a case report
Source: Front Immunol. 2023 Dec 20;14:1277810. doi: 10.3389/fimmu.2023.1277810 (PMC10765511; doi:10.3389/fimmu.2023.1277810)
Supplement: Supplementary file 1 [file Table_1.docx]

Supplementary Materials

Making “cold” tumors “hot”- Radiotherapy remodels the tumor immune microenvironment of pancreatic cancer to benefit from Immunotherapy: A case report.

Fan Tong^1,2^ †, Yi Sun^1^ †, Yahui Zhu^1^, Huizi Sha^1^, Jiayao Ni^1,2^, Liang Qi^1^, Qing Gu^3^, Chan Zhu^4^, Wenjing Xi^4^, Baorui Liu^1*^, Weiwei Kong^1*^, Juan Du^1*^

*** Correspondence:** Baorui Liu email: [baoruiliu@nju.edu.cn](mailto:baoruiliu@nju.edu.cn), Weiwei Kong email: [kongvv@126.com](mailto:kongvv@126.com) and Juan Du email: [dujuanglyy@163.com](mailto:dujuanglyy@163.com)

†These authors contributed equally to this work.

**Supplementary Table 1** Immune scores of gene expression analysis at the RNA level on M2 hepatic metastasis prior and post treatment, respectively.

| **signature_type** | **signature_name** | **prior treatment** | **post treatment** |
| --- | --- | --- | --- |
| Inhibitory Immune Mechanisms | B7-H3 | 9.56 | 9.42 |
| Inhibitory Immune Mechanisms | IDO1 | 3.32 | 6.77 |
| Inhibitory Immune Mechanisms | PD-L1 | 3.81 | 5.58 |
| Inhibitory Immune Mechanisms | TGF-Beta | 7.69 | 8.61 |
| Inhibitory Immune Signaling | ARG1 | 11.21 | 13.5 |
| Inhibitory Immune Signaling | CTLA4 | 2.58 | 5.32 |
| Inhibitory Immune Signaling | IL10 | 3.46 | 4.52 |
| Inhibitory Immune Signaling | NOS2 | 2.58 | 3.81 |
| Inhibitory Immune Signaling | PD-1 | 2.58 | 3.81 |
| Inhibitory Immune Signaling | PD-L2 | 5.29 | 7.08 |
| Inhibitory Immune Signaling | TIGIT | 2.58 | 6.19 |
| Immune cell abundance | B Cells | 2.8 | 3.92 |
| Immune cell abundance | CD45 | 6.02 | 8.69 |
| Immune cell abundance | CD8 T Cells | 4.13 | 7.48 |
| Immune cell abundance | Cytotoxic Cells | 4.08 | 7.09 |
| Immune cell abundance | DC | 6.62 | 7.84 |
| Immune cell abundance | Exhausted CD8 | 3.99 | 5.95 |
| Immune cell abundance | Macrophages | 7.34 | 9.4 |
| Immune cell abundance | Mast Cells | 3.84 | 5.66 |
| Immune cell abundance | Neutrophils | 4.61 | 6.28 |
| Immune cell abundance | NK CD56dim | 2.83 | 4.37 |
| Immune cell abundance | NK Cells | 2.58 | 4.39 |
| Immune cell abundance | T Cells | 3.87 | 6.81 |
| Immune cell abundance | TH1 Cells | 3.17 | 5.75 |
| Immune cell abundance | Treg | 3.32 | 5.36 |
| Immune cell abundance | Macrophages M1 | 4.48 | 6.52 |
| Immune cell abundance | Macrophages M2 | 6.05 | 8.07 |
| Tumor Immunogenicity | Immunoproteasome | 8.35 | 8.99 |
| Tumor Immunogenicity | MAGEs | 2.58 | 3.81 |
| Anti-Tumor Immune Activity | TIS | -0.09 | 0.48 |
| Anti-Tumor Immune Activity | Cytotoxicity | 3.61 | 6.5 |
| Anti-Tumor Immune Activity | IFN Gamma | 5.29 | 8.51 |
| Anti-Tumor Immune Activity | IFN Downstream | 7.61 | 9 |
| Anti-Tumor Immune Activity | Lymphoid | 4.02 | 6.58 |
| Anti-Tumor Immune Activity | MHC2 | 7.86 | 9.74 |
| Inhibitory Immune Signaling | Inflammatory Chemokines | 5.62 | 6.51 |
| FCR | FCR | 8.25 | 9.63 |
| 289Signature | CYT.Score | 5.28 | 8.76 |
| 289Signature | T cell Markers | 6.71 | 8.74 |
| 289Signature | Cytotoxic T Lymphocyte Level | 4.43 | 7.64 |
| 289Signature | T-effector | 4.03 | 6.33 |
| 289Signature | Immune Signature | 4.32 | 7.78 |
| Total.TILs.Score | Total.TILs.Score | 3.93 | 5.77 |

**Selection of immune-related genes**

Genes were grouped into 16 immune cell type meta genes and 27 immune functions according to the manufacturer’s designation.

Marker genes of 14 immune cell types, including B-cells, dendritic cells (DCs), macrophages, T-cells, regulatory T cells (Tregs), CD8 T cells, exhausted CD8, neutrophils, mast cells, cytotoxic cells, natural killer (NK) cells, NK CD56dim cells, CD45, and Th1 cells were retrieved from the method previously reported (1-3). We further divided the macrophages into macrophages M1 and macrophages M2 according to the previous reports (4, 5). All TIME cell infiltration scores were calculated as arithmetic mean of the constituent genes (1).

The metagene scores were calculated as the geometric mean expression of the member genes(6). In additation, We also examined 7 previously published prognostic and immune therapy response predictive meta genes , including (1) CYT (cytolytic activity) score; (2) cytotoxic T Lymphocyte Level;(3) Immune Signature;(4) Teff (T-effector) score;(5)T cell Markers; (6) Total.TILs.Score (tumor infiltrating lymphocytes) score;(7) TIS. The CYT score of each sample can be evaluated based on the geometric mean of the product of PRF1 and GZMA genes (7); TIS score was calculated as a weighted linear average of the constituent genes (8, 9); the remaining five TIME signatures were calculated as arithmetic mean of the corresponding gene (1) .

**Supplementary Table : TME 289 gene list**

| ABCF1 | CD44 | FCGR1A | IL21R | NFKBIA | TIE1 |
| --- | --- | --- | --- | --- | --- |
| ADM | CD47 | FCGR2B | IL2RA | NKG7 | TIGIT |
| ADORA2A | CD48 | FCRL2 | IL2RB | NOS2 | TLR3 |
| AKT1 | CD6 | FGF13 | IL2RG | NT5E | TLR7 |
| ANGPT2 | CD68 | FOXP3 | IL4 | OAS1 | TLR8 |
| ARG1 | CD69 | FPR1 | IL6 | OAS2 | TLR9 |
| ATM | CD70 | FUT4 | IL7R | OAS3 | TNF |
| AXL | CD74 | G6PD | IRF1 | PDCD1 | TNFRSF14 |
| BCL2 | CD79A | GBP1 | IRF4 | PDCD1LG2 | TNFRSF17 |
| BIRC5 | CD79B | GNLY | IRF9 | PDGFA | TNFRSF18 |
| BLK | CD80 | GUSB | ISG15 | PDGFB | TNFRSF1A |
| BLM | CD84 | GZMA | ITGA1 | PECAM1 | TNFRSF1B |
| BRCA1 | CD86 | GZMB | ITGAE | PIK3CA | TNFRSF4 |
| BRCA2 | CD8A | GZMH | ITGAL | PIK3CD | TNFRSF9 |
| BRIP1 | CD8B | GZMK | ITGAM | PMS2 | TNFSF10 |
| BTLA | CDKN2A | HAVCR2 | ITGAX | PNOC | TNFSF13B |
| C1QA | CEACAM3 | HDC | ITGB2 | POLR2A | TNFSF18 |
| C1QB | CMKLR1 | HERC6 | KIR2DL3 | PRF1 | TNFSF4 |
| CCL13 | CPA3 | HIF1A | KIR3DL1 | PSMB10 | TNFSF9 |
| CCL18 | CSF1R | HLA-DMA | KIR3DL2 | PSMB9 | TRAT1 |
| CCL2 | CSF2 | HLA-DMB | KLRB1 | PTEN | TWIST1 |
| CCL20 | CSF2RB | HLA-DOA | KLRD1 | PTGER4 | VCAM1 |
| CCL21 | CSF3R | HLA-DOB | KLRK1 | PTGS2 | VEGFA |
| CCL22 | CTAG1B | HLA-DPA1 | LAG3 | PTPN11 | VTCN1 |
| CCL4 | CTLA4 | HLA-DQA2 | LCK | PTPRC | ZAP70 |
| CCL5 | CTSS | HLA-DRA | LILRB2 | PVR | ZEB1 |
| CCL7 | CTSW | HSD11B1 | LY9 | RAD51 | CXCL2 |
| CCND1 | CX3CL1 | ICAM1 | LYZ | RB1 | FCGR3B |
| CCR2 | CX3CR1 | ICOS | MAGEA1 | RORC | GZMM |
| CCR4 | CXCL1 | ICOSLG | MAGEA12 | RUNX3 | HLA-DQA1 |
| CCR5 | CXCL10 | IDO1 | MAGEA4 | S100A12 | HLA-DRB1 |
| CD14 | CXCL11 | IFI27 | MAGEC2 | S100A8 | HLA-E |
| CD163 | CXCL12 | IFI35 | MELK | S100A9 | OAZ1 |
| CD19 | CXCL13 | IFI6 | MKI67 | SDHA | PF4 |
| CD1C | CXCL5 | IFIH1 | MLANA | SELL | PRR5 |
| CD2 | CXCL8 | IFIT1 | MLH1 | SH2D1A | STK11IP |
| CD209 | CXCL9 | IFIT2 | MMP9 | SIGLEC5 | TBC1D10B |
| CD244 | CXCR2 | IFIT3 | MRC1 | SLAMF7 | TPSAB1 |
| CD247 | CXCR3 | IFITM1 | MS4A1 | SNAI1 | UBB |
| CD27 | CXCR4 | IFITM2 | MS4A2 | SPIB |  |
| CD274 | CXCR6 | IFNG | MS4A4A | STAT1 |  |
| CD276 | CYBB | IL10 | MSH2 | STAT3 |  |
| CD28 | DLL4 | IL10RA | MSH6 | STAT4 |  |
| CD38 | EGFR | IL12RB2 | MTOR | TAP1 |  |
| CD3D | EIF2AK2 | IL15 | MX1 | TBP |  |
| CD3E | ENTPD1 | IL17A | MYC | TBX21 |  |
| CD3G | EOMES | IL18 | NBN | TCL1A |  |
| CD4 | FAS | IL1A | NCAM1 | TDO2 |  |
| CD40 | FASLG | IL1B | NCR1 | TFRC |  |
| CD40LG | FCAR | IL2 | NECTIN2 | TGFB1 |  |

**Supplementary Table: Cell type gene list**

| **Cell type** | **Gene list** |
| --- | --- |
| T cells | CD3D、CD3E、CD3G、CD6、SH2D1A、TRAT1 |
| B cells | BLK、CD19、FCRL2、MS4A1、PNOC、SPIB、TCL1A、TNFRSF17 |
| Mast cells | CPA3、HDC、MS4A2 |
| DC | CCL13、CD209、HSD11B1 |
| Macrophages | CD163、CD68、CD84、MS4A4A |
| Neutrophils | CEACAM3、CSF3R、FCAR、FPR1、S100A12、SIGLEC5 |
| Cytotoxic cells | CTSW、GNLY、GZMA/B/H、KLRB1、KLRD1、KLRK1、NKG7、PRF1 |
| Exhausted CD8 | PTGER4、LAG3、EOMES、CD244 |
| NK CD56 cell | KIR3DL1/2/3、IL21R |
| CD8 T cell | CD8B、CD8A |
| CD45 cell | PTPRC |
| Th1 cell | TBX21 |
| NK cell | NCR1 |
| Treg cell | FOXP3 |

**Reference:**

1. Danaher P, Warren S, Dennis L, D'Amico L, White A, Disis ML, et al. Gene expression markers of Tumor Infiltrating Leukocytes. Journal For Immunotherapy of Cancer. 2017;5:18.

2. Bindea G, Mlecnik B, Tosolini M, Kirilovsky A, Waldner M, Obenauf AC, et al. Spatiotemporal dynamics of intratumoral immune cells reveal the immune landscape in human cancer. Immunity. 2013;39(4):782-95.

3. Newman AM, Liu CL, Green MR, Gentles AJ, Feng W, Xu Y, et al. Robust enumeration of cell subsets from tissue expression profiles. Nat Methods. 2015;12(5):453-7.

4. Germano G, Frapolli R, Belgiovine C, Anselmo A, Pesce S, Liguori M, et al. Role of macrophage targeting in the antitumor activity of trabectedin. Cancer Cell. 2013;23(2):249-62.

5. Mosser DM, Edwards JP. Exploring the full spectrum of macrophage activation. Nat Rev Immunol. 2008;8(12):958-69.

6. Zheng J, Yang M, Shao J, Miao Y, Han J, Du J. Chemokine receptor CX3CR1 contributes to macrophage survival in tumor metastasis. Mol Cancer. 2013;12(1):141.

7. Vitiello GA, Bowler TG, Liu M, Medina BD, Zhang JQ, Param NJ, et al. Differential immune profiles distinguish the mutational subtypes of gastrointestinal stromal tumor. J Clin Invest. 2019;129(5):1863-77.

8. Ayers M, Lunceford J, Nebozhyn M, Murphy E, Loboda A, Kaufman DR, et al. IFN-γ-related mRNA profile predicts clinical response to PD-1 blockade. J Clin Invest. 2017;127(8):2930-40.

9. Damotte D, Warren S, Arrondeau J, Boudou-Rouquette P, Mansuet-Lupo A, Biton J, et al. The tumor inflammation signature (TIS) is associated with anti-PD-1 treatment benefit in the CERTIM pan-cancer cohort. J Transl Med. 2019;17(1):357.
